# The Effect of Carvacrol on Serum Cytokines and Endothelin Levels of Ovalbumin Sensitized Guinea-Pigs

**Published:** 2013-04

**Authors:** Sediqeh Jalali, Mohammad Hossein Boskabady, Ali Haeri Rohani, Akram Eidi

**Affiliations:** 1 Department of Biology, Science and Research Branch, Islamic Azad University, Tehran, Iran; 2 Department of Physiology, School of Medicine, and Pharmaceutical Research Centre, Iran

**Keywords:** Asthma, Carvacrol, Cytokines, Dexamethasone, Sensitization

## Abstract

***Objective(s):*** Different pharmacological effects of carvacrol including relaxant effect, its inhibitory effect on muscarinic and histamine (H_1_) and stimulatory effect on β-adrenoceptors have been demonstrated on guinea pig tracheal chains in previous studies. In the present study, the effect of carvacrol on blood IL-4, IFN- γ and endothelin levels of sensitized guinea pigs is examined.

***Materials and Methods:*** Five groups of guinea pigs sensitized to ovalbumin (OA) were given pure drinking water (group S), drinking water containing three concentrations of carvacrol (40, 80 and 160 µg/ml)) and dexamethasone. The blood IL-4, IFN- γ and endothelin levels of sensitized and control guinea pigs were evaluated (n=6, for all groups).

***Results:*** Blood IL-4 and IFN-γ levels (*P<*0.001 for both cases) as well as endothelin (*P<*0.01) were increased but IFN-γ/IL-4 ratio decreased (*P<*0.05) in sensitized animals compared to controls. The treatment of S animals by dexamethasone (*P<*0.01) and two higher concentrations of carvacrol (*P<*0.001 for both cases) significantly decreased IL-4 level. The treatment of S animals with dexamethasone did not changed IFN-γ levels but treatment with high concentration of carvacrol significantly increased its level (*P<*0.001). In addition, IFN-γ/IL-4 ratio was significantly increased in S groups, who were treated with dexamethasone (*P<*0.05) and two higher concentrations of carvacrol (*P<*0.001 for both cases). Treatment of S animals by dexamethasone (*P<*0.01) and all concentrations of carvacrol also significantly decreased endothelin level (*P<*0.01 to *P<*0.001).

***Conclusion:*** The results show that carvacrol causes the reduction of IL-4 and endothelin, but it increases IFN-γ and IFN-γ/IL-4 ratio in the blood of sensitized guinea pigs. The results also suggest more specific effect of carvacrol compared to dexamethasone due to the absence of the effect of later on IFN-γ.

## Introduction 

The main characteristic of asthma is airway inflammation (1). Different inflammatory cells are involved in the pathogenesis of airway inflammation in asthma (2). The immunologic features of asthma is a balance shift from T helper 1 (Th1) to Th2. It is evident that Th2 is activated in asthma disease, and its mediators cause many features of asthma such as airway inflammation, mucus secretion and airway hyperresponsiveness (3). Th1 has proved to inhibit Th_2_ responses and thus one goal of asthma therapy should be increasing the balance of Th1/Th2 (4). Therefore, modulation of T cell responses toward Th1 or Th2 can change the fate of an immune response in favour of a wanted situation. In fact, the treatment goal for asthma disease is anti-inflammatory or preventive drugs but other types of drugs used for the treatment of this disease are bronchodilatory or relieving agents. 

Carvacrol or cymophenol, C_6_H_3_CH_3_(OH)(C_3_H_7_) is one of the main constituent of everal medicinal plants including *Zataria multiflora Bois*s (5) which have therapeutic effect on respiratory disease (5-7).

All plants containing carvacrol have shown relaxant effect on different smooth muscles including trachea (8-14). The relaxant effect of essential oil from *Carum copticum* on tracheal chains has been shown to be mainly due to its fraction 2, which is suggested to be carvacrol (15). The relaxant effect of carvacrol on tracheal chains has also been observed in another study (16). These studies indicate that carvacrol or plant containing this chemical material may affect asthma therapy as a bronchodilator or relieving drug. 

To examine one aspect of anti-inflammatory effect of carvacrol and its potential preventive effect in inflammatory disorders such as asthma, in the present study, the effect of carvacrol on IL-4 and increased IFN-γ and IFN-γ/IL-4 ratio as an index of Th1/Th2 activity on sensitized guinea pigs has been examined.

## Materials and Methods

*Animal sensitisation and animal groups*

Animals were sensitized to OA according the method described previously (17, 18). Briefly, guinea pigs were sensitized to 10 mg OA (Sigma Chemical Ltd, UK) and 100 mg Al (OH)_3_ dissolved in 1 ml saline i.p. One week later they were given 2 mg OA and 100 mg Al (OH)_3_ dissolved in 1 ml saline i.p. as a booster dose. From day 14 sensitized animals were exposed to an aerosol of 4% OA for 18±1 days, 5 min daily. The aerosol was administered in a closed chamber, dimensions 30 x 20 x 20 cm. Control animals were treated similarly but saline was used instead of OA solution. The study was approved by the ethical committee of the ashhad University of Medical Sciences.

The study was performed in control animals (group C, treated the same as sensitized group, but normal saline was used instead of OA and they were given pure drinking water) and five different groups of sensitized animals which were pure given drinking water (group S, an animal model of asthma) or drinking water containing treatment agents during sensitization period as follows (n=6 for each group);

1. 50 µg/ml dexamethasone (group S+D)

2. 40 µg/ml carvacrol (group S+C1)

3. 80 µg/ml carvacrol (group S+C2)

4. 160 µg/ml carvacrol (group S+C3)

***Measurement of blood IL-4 and IFN- γ*** ***levels***

Five ml of peripheral blood was obtained immediately after sacrificing the animals and placed at room temperature for 1 hr. The samples were then centrifuged at 2500 rpm at 4°C for 10 min. The supernatant was collected and immediately stored at 70° C until analyzed. Finally, blood IL-4 and IFN- γ levels were measured using Elisa sandwich (Ab Sandwich) method. The Elisa kits were purchased from Bender Medsystem Sakhte, Austria. The ratio of IFN-γ/IL4 as an index of Th1/Th2 was also calculated.

*Measurement of blood endothelin levels*

Five ml peripheral blood was obtained immediately after sacrificing the animals and placed at room temperature for 1 hr. The samples were then centrifuged at 2500 rpm at 4°C for 10 min. The supernatant was collected and immediately stored at 70°C until analyzed. Blood endothelin was measured using the enzyme-linked immunosorbent assay (ELISA) Sandwich method according to the manufacturer’s instructions, (IBL’s ET-1 Assay Kit, Code No. 27165).

*Statistical analysis*

The data were quoted as mean±SEM. According to the Kolmogorov Smirnov test, the data had normal distribution. The data of sensitized group were compared with the control guinea pigs using unpaired t- test. The data of treated groups was also compared with sensitized guinea pigs using unpaired “t” test. The data of three groups of animals treated with carvacrol were compared with each other using one way ANOVA through Tukey Kermar post hoc test. Significance was accepted at *P<*0.05.

## Results

***The serum levels of cytokine, endothelin and Th1/Th2 balance***

**Figure 1.**
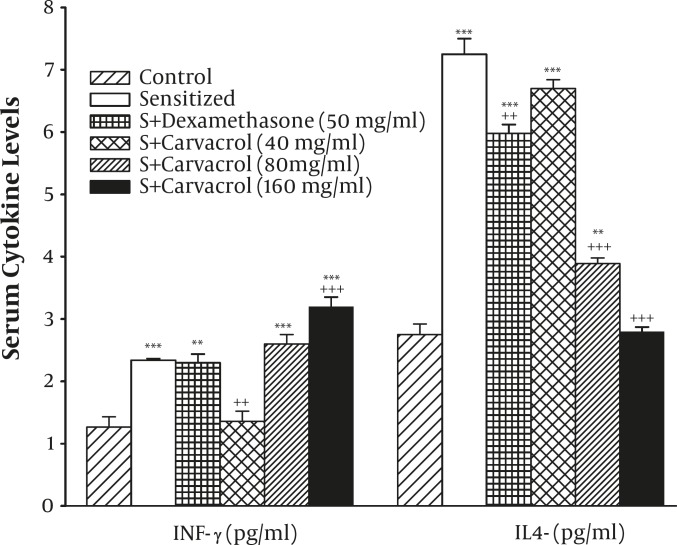
The levels of serum INF-γ and IL-4 in control (C), sensitized (S), S treated with dexamethasone and three concentrations of carvacrol guinea pigs (for each group, n=6). Statistical differences between the control and other groups: **; P<0.01, ***; P<0.001. Statistical differences between treated animals vs sensitized group: +: P<0.05, ++: P<0.01, +++: P<0.001

In the sensitized group, the serum levels of IL-4 (7.25±0.25) and IFN-γ (2.34±0.04) in group S were significantly higher than that of the group C (2.75±1.17 and 1.26±0.17 for IL-4 and IFN-γ respectively), (*P<*0.001 for both cases, Figure 1).

The treatment by two higher concentrations of carvacrol (*P<*0.001 for both cases) and dexamethasone (*P<*0.01) caused significant reduction in IL-4. However, the level IFN-γ was increased in the treated group with the high concentration (160 µg/ml) of carvacrol (*P<*0.001, Figure 1). 

**Figure 2:**
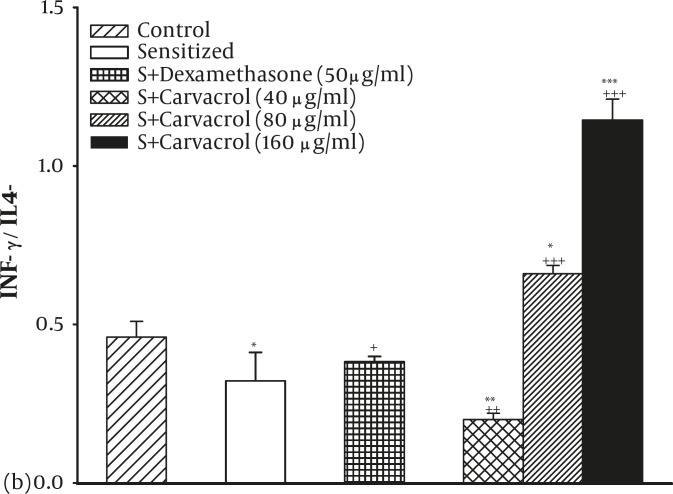
The ratio of serum INF-γ/IL-4 (Th1/Th2 balance) in control (C), sensitized (S), S treated with dexamethasone and three concentrations of Carvacrol guinea pigs (for each group, n=6). Statistical differences between the control and other groups: *; P<0.05, **; P<0.01. Statistical differences between treated animals vs sensitized group: +: P<0.05, ++: P<0.01, +++: P<0.001

The IFN-γ/IL4 ratio (Th1/Th2 balance) was also significantly decreased in the sensitized group compared to the control group (*P<*0.05, Figure 2). However, the treatment of sensitized animals by dexamethasone (*P<*0.05) and two higher concentration of carvacrol (*P<*0.001 for both cases) caused significant increase in Th1/Th2 balance (Figure 2).

**Figure 3:**
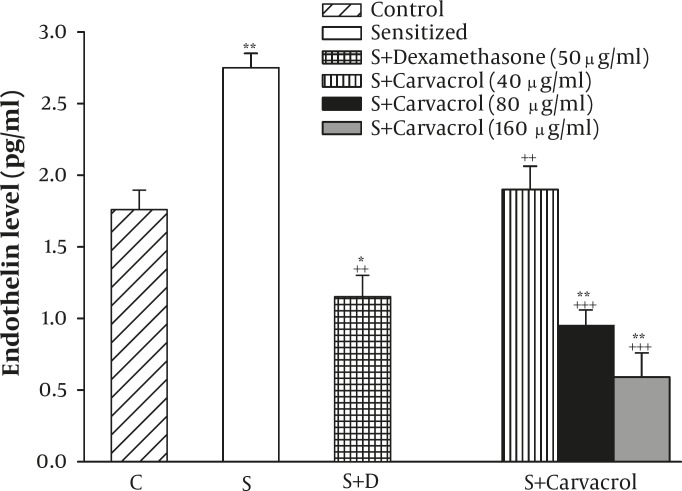
The levels of serum endothelin in control (C), sensitized (S), S treated with dexamethasone and three concentrations of carvacrol guinea pigs (for each group, n=6). Statistical differences between the control and other groups: **; P<0.01, ***; P<0.001. Statistical differences between treated animals vs sensitized group: ++: P<0.01, +++: P<0.001, +++: P<0.001

In addition, the serum level of endothelin (2.75±0.10) in group S were significantly higher than group C (1.76±0.14), (*P<*0.01, Figure 3). The treatment of sensitized animals by all concentrations of carvacrol caused significant reduction in serum endothelin level (*P<*0.01 to *P<*0.001, Figure 3). 

***Differences in the serum levels of cytokine, endotheline and Th1/Th2 balance between dexamethasone and carvacrol treated groups***

The effect of the low concentration of carvacrol (40 µg/ml) on IL-4, IFN-γ, IFN-γ/IL4 ratio and endothelin was significantly lower than the effect of dexamethasone (*P<*0.05 to *P<*0.001, Table 1). However, the effects of two higher concentrations of carvacrol (80 and 160 µg/ ml (l)) on IL-4, IFN-γ, IFN-γ/IL4 ratio and the effect of its highest concentration on endothelin were significantly higher than the effect of dexamethasone (*P<*0.01 to *P<*0.001, Table 1). 

**Table 1:** The values of the serum cytokine levels of the control guinea pigs sensitized animals (S), S treated with three concentrations of carvacrol (S+C1, S+C2 and S+C3) and S treated by dexamethasone (S+D), (for each group, n=6)

Cytokine	Control	S	S+D	S+C1	S+C2	S+C3
IL-4	2.75±0.17	7.25±0.25	5.98±0.14	6.70±0.14 *	3.80±0.09 *** +++	2.80±0.07 *** +++ †††
IFN-γ	1.26±0.17	2.34±0.04	2.30±0.14	1.36±0.16 **	2.60±0.15 +++	3.20±0.15 ** +++
IFN-γ/IL-4	0.46±0.06	0.32±0.01	0.38±0.02	0.20±0.02 ***	0.67±0.03 *** +++	1.46±0.07 *** +++ †††
Endotheline	1.76±0.14	2.75±0.10	1.152±0.15	1.90±0.16 **	0.95±0.11 ++	0.59±0.17 ** +++ ††

**The values are presented as mean±SEM. Three concentrations of the extract were 40 (S+C1), 80 (S+C2) and 160 µg/ml (S+C3) and that of dexamethasone was 50 μg/ml.**

Statistical significance for the difference between the data of S+D vs S+C1, S+C2 and S+C3: *; ***P<*** 0.05, **; ***P<*** 0.01, ***; ***P<*** **0.001. The statistical comparisons were made using unpaired t test.**

**Statistical significance for the difference between the data of S+C3 vs S+C1 vs S+C2: ++;** ***P<*** **0.01, +++;** ***P<*** **0.001. Statistical significance for the difference between the data of S+C2 vs S+C3: **††**; *P<*0.01, **†††**; *P<*0.001. The statistical comparisons were made using ANOVA with Tukey- Kramer multiple pot test**

***Differences in serum levels of cytokine, endotheline and Th1/Th2balance between three concentrations of carvacrol***

The effect of two higher concentrations of carvacrol (80 and 160 µg/ ml) on IL-4, IFN-γ, IFN-γ/IL4 ratio and endotheline was significantly higher than the effect of its low concentration (*P<*0.01 to *P<*0.001, Table 1). In addition, the effect of the high concentration of carvacrol on IL-4, IFN-γ/IL4 ratio and endothelin was significantly higher than the effect of its middle concentration (*P<*0.01 *P<*0.001, Table 1).

## Discussion

In the present study, the effect of carvacrol, as the constituent of different medicinal plants including *Zataria multiflora* and *Tymus volgaris* was examined on IL-4, IFN-γ, IFN-γ/IL-4 ratio as an index of Th1/Th2 activity and enothelin on sensitized guinea pigs. The results of the present study showed increased serum IL-4, IFN-γ and endotheline levels; but, IFN-γ/IL-4 ratio decreased in sensitized animals compared to the controls. 

The treatment of sensitized animals by two higher concentrations of carvacrol significantly decreased IL-4 and endothelin levels. In addition, treatment by the high concentration of carvacrol significantly increased IFN-γ level and IFN-γ/IL-4 ratio in sensitized group. Dexamethasone treatment also caused significant reduction in IL-4 and endothelin, but did not affect IFN-γ level and IFN-γ/IL-4 ratio. The effect of two higher concentrations of carvacrol (80 and 160 µg/ml) on IL-4, IFN-γ, endotheline and IFN-γ/IL4 ratio was significantly higher than the effect of dexamethasone.

Airway inflammation occurring in asthma is regulated by the balance of Th1 /Th2 cells (3, 4). Th2 cells release IL-4, which increases the activity of B cells and releases IgE. This, in turn, can stimulate the pro-inflammatory responses mediated by inflammatory cells such as eosinophils. However, Th1 cells can decrease the activities of Th2 and regulate the anti- inflammatory response (4). 

Th1 cells release interleukin 2 (IL-2) and IFN-γ, but Th2 cells produce IL-4 and IL-10 (19). Therefore, the Th2 response is orchestrating humoral immunity but Th1 orchestrate cell mediated immunity (CMI) to overcome intracellular bacteria, viruses and cancers. An inappropriate change in Th1/Th2 balance can cause hyper or hypo-reactivity result in immune-pathological diseases such as allergy, autoimmunity or cancer.

Therefore, the results of the present study suggest that carvacrol has inhibitory effect on IL-4 but enhances the production of IFN- γ, indicating the inhibitory effect on Th2 and stimulatory effect on Th1 helper cells. The results of the present study also show raise in the ratio of IFN-γ to IL-4 due to carvacrol treatment in sensitized guinea pigs. However, serum IFN-γ level has also increased in sensitized guinea pigs treated with the last concentration of carvacrol while dexamethasone has not affected it. These results indicate that carvacrol causes inhibitory effect on Th2 similar to dexamethasone but has a stimulatory effect on Th1 helper cells which is different with the effect of dexamethasone. Therefore, the results of the present study show that in inflammatory disorders associated with decreasing the balance of Th1/Th2, carvacrol may show even more therapeutic values compared to dexamethasone.

The effect of endothelin on asthmatic inflammation, proliferation of bronchial smooth muscle cells and subepithelial fibrosis has been suggested, that leads to airway remodeling and severe bronchial hyperreactivity (20). The effect of endothelin in bronchoconstriction, mucus secrection, and plasma exudation (21, 22) has also been observed. The elevated levels of endothelin in bronchoalveolar fluid in asthmatics and its correlation with the severity of disease (23), its increased concentration in exhaled breath condensatein of asthmatic patients has been observed (24),supporting the findings of the present study. The reduction of the level of endothelin in the serum of sensitized guinea pigs treated with carvacrol indicates anti-inflammatory effect of this agent and its possible preventive effect on asthma disease. The similar effect of carvacrol with dexamethasone on sensitized animals supports the anti-inflammatory effect of carvacrol. 

The results also show the concentration-dependent effect of carvacrol on IL-4, IFN-γ and endotheline level as well as IFN-γ/IL-4 ratio. The greater effect of two higher concentration of carvacrol compared to dexamethasone suggests that the effect of carvacrol is more potent than dexamethasone on IL-4, IFN-γ and endotheline level as well as IFN-γ/IL-4 ratio in sensitized guinea pigs at concentrations used.

Our previous study showed the potent relaxant effect of the extract of other plant containing carvacrol (*Tymus volgaris*) (11) and carvacrol itself (16) on tracheal chains, the stimulatory effect of the plant on ß_2_-adrnoceptors (25) and the inhibitory effect of the plant and carvacrol on histamine (H_1_) receptors (26). In addition, the inhibitory effect of carvacrol (27) and the extract of *Zataria multiflora *(28) on muscrinic receptors of tracheal smooth muscle were also documented. 

## conclusion

The results of this study showed that carvacrol caused the reduction of IL-4 and endothelin but increased IFN-γ and IFN-γ/IL-4 ratio in the blood of sensitized guinea pigs. The effect of carvacrol was more specific compared to dexamethasone due to the absence of the effect of later on IFN-γ. The results also suggested that the effect of carvacrol was more potent than dexamethasone on IL-4, IFN-γ and endotheline level as well as IFN-γ/IL-4 ratio in sensitized guinea pigs at concentrations used.
